# Risk Factors of Chronic Kidney Disease Progression: Between Old and New Concepts

**DOI:** 10.3390/jcm13030678

**Published:** 2024-01-24

**Authors:** Francesca Mallamaci, Giovanni Tripepi

**Affiliations:** 1 Nephrology, Dialysis and Transplantation Unit, Grande Ospedale Metropolitano, Bianchi-Melacrino-Morelli (BMM), 89124 Reggio Calabria, Italy; 2 Research Unit of Clinical Epidemiology of Reggio Calabria, Institute of Clinical Physiology (IFC), National Research Council (CNR), 89124 Reggio Calabria, Italy

**Keywords:** chronic kidney disease, aging, hypertension, obesity, salt intake, sympathetic overactivity

## Abstract

Chronic kidney disease (CKD) is a condition characterized by the gradual loss of kidney function over time and it is a worldwide health issue. The estimated frequency of CKD is 10% of the world’s population, but it varies greatly on a global scale. In absolute terms, the staggering number of subjects affected by various degrees of CKD is 850,000,000, and 85% of them are in low- to middle-income countries. The most important risk factors for chronic kidney disease are age, arterial hypertension, diabetes, obesity, proteinuria, dyslipidemia, and environmental risk factors such as dietary salt intake and a more recently investigated agent: pollution. In this narrative review, we will focus by choice just on some risk factors such as age, which is the most important non-modifiable risk factor, and among modifiable risk factors, we will focus on hypertension, salt intake, obesity, and sympathetic overactivity.

## 1. Introduction

Chronic kidney disease (CKD) is a progressively degenerative condition characterized by the gradual deterioration of renal function, representing a significant and pressing global health issue. CKD affects about 10% of the general population worldwide [1]. Yet, when interpreted in absolute figures, the profound scale of individuals affected by various stages of CKD amounts to 850 million [1], with a substantial 85% concentrated in low- to middle-income countries. Almost innumerable determinants contribute to the development and progression of chronic kidney disease, including age, hypertension, genetic factors, diabetes, obesity, proteinuria, dyslipidemia, and environmental factors such as dietary salt intake [2,3,4]. Notably, recent research has identified pollution as an additional risk factor [5]. More recently, an escalating body of evidence underscores the implications of climate change and pollution as emergent detrimental factors to overall public health, particularly concerning kidney diseases [5]. The list of risk factors for the progression is even more complex, and many of them are intimately interrelated. To start with ascertaining the causes of the CKD epidemics, a deep analysis of the population’s composition is imperative, mainly with stratification by age. The demography is visually delineated with an age-specific “age pyramid.” The Italian population was recorded at 59 million in 2012, which is expected to exceed 61 million in 2056. In 2012, the proportions of individuals in Italy aged above 65 (classified as young elderly) and over 80 years were 22% and 7%, respectively. Projections for 2056 estimate these figures to rise to 34% and 10%. According to the Chares study [6], the prevalence of kidney disease within the demographic cohort aged 60 years and older is around 26%. Noteworthy is the fact that the risk for end-stage kidney disease (ESKD) is relatively low within the pediatric age group and remains low until age 40. Subsequently, a marked exponential escalation in risk ensues. Upon reaching 65, the probability of dialysis therapy reaches 3%, implying that 1 out of every 33 individuals within this age cohort is undergoing dialysis treatment. As individuals advance to 80 years of age, this risk elevates to 5%, denoting that 1 in 20 subjects in this age group will require dialysis care. All these considerations unequivocally indicate that CKD is not a condition confined to a specific niche; rather, its prevalence surpasses that of diabetes. Apart from age, the above-mentioned risk factors are modifiable, and focusing on modifiable risk factors for the progression of CKD is crucial for improving patient outcomes, optimizing resource allocation, developing preventive measures, enhancing cost-efficiency, making a public health impact, and driving research and innovation in kidney disease management. Last but not least, it is of paramount importance to point out that CKD is the fastest-growing chronic disease and is one of the most important causes of mortality. In fact, in the early nineties of the past century, CKD was the 36th cause of death among chronic diseases, and after 20 years, it is in 19th place in this macabre ranking, and forecasts for the coming decades are even worse. Indeed, the progression of CKD involves several interconnected mechanisms. We will now begin to focus on some of them, starting with the most prominent and non-modifiable, such as age. This narrative review will focus on some specific risk factors for the progression of CKD, such as age, hypertension and salt intake, obesity, and sympathetic hyperactivity. By managing these risk factors, healthcare providers can potentially slow the disease’s advancement and mitigate complications, ultimately improving patients’ quality of life. Indeed, understanding the risk factors mentioned above for CKD progression would enable the development of preventive strategies, lifestyle modifications, medications, or other interventions that aim to mitigate or eliminate the risk factors, potentially preventing the onset or progression of CKD.

## 2. Aging and CKD

As the global population ages, the prevalence of CKD continues to rise [5,6,7], prompting a critical examination of the intricate relationship between aging and renal health. The age-dependent increase in CKD can be primarily attributed to the escalating prevalence of risk factors for CKD, including diabetes, hypertension, obesity, and cardiovascular disease as well as the action of some effect modifiers such as smoking [8]. Additionally, the expansion of the range of GFR to define CKD substantially contributes to this trend [9]. Aging is accompanied by a myriad of physiological and pathophysiological changes, including alterations in renal structure and function. A decline in renal mass, a reduced number of nephrons, and impaired renal blood flow are common age-related changes that can contribute to the development and progression of CKD. Additionally, aging is associated with increased susceptibility to oxidative stress, inflammation, and fibrosis, all of which are implicated in the pathogenesis of CKD [10]. The aging process exerts a profound impact on renal hemodynamics, with alterations in blood pressure regulation and renal autoregulation [11]. These changes can lead to increased glomerular pressure, proteinuria, and a heightened risk of kidney injury. Understanding the hemodynamic consequences of aging is crucial for comprehending the accelerated progression of CKD observed in elderly individuals. Cellular senescence, a hallmark of aging, has been implicated in the progression of CKD [12]. Senescent cells accumulate in the kidneys over time, releasing pro-inflammatory cytokines and contributing to tissue fibrosis. This senescence-associated secretory phenotype may exacerbate inflammation and promote the transformation of healthy renal tissue into a pro-fibrotic environment. Aging is often accompanied by alterations in metabolic pathways, including insulin resistance and dyslipidemia, which can further contribute to the progression of CKD [13,14,15,16,17]. Metabolic derangements may impair renal function through mechanisms such as glomerular hyperfiltration and increased oxidative stress. Understanding the impact of aging on CKD progression has important clinical implications. Elderly individuals with CKD often face unique challenges, including the management of comorbidities and potential drug interactions [18,19]. Tailored therapeutic approaches that account for both age-related changes and CKD-specific factors are essential to optimize patient outcomes.

There is a scarcity of evidence-based guidelines and recommendations for effectively treating CKD in the elderly population. Addressing geriatric considerations such as frailty, quality of life, life expectancy, pharmacokinetics, pharmacodynamics of drugs, and treatment complications is crucial in formulating a comprehensive approach to CKD management in older individuals. Factors leading to acute kidney injuries, such as nephrotoxic antibiotics, radio-contrast exposure, and certain medication combinations, including angiotensin-converting enzyme inhibitors (ACEIs) and angiotensin receptor blockers (ARBs), as well as nonsteroidal anti-inflammatory drugs (NSAIDs) and diuretics, must be avoided [20,21]. Adopting a general strategy for CKD management, the distinctive challenges of managing CKD in the elderly are underscored. The overarching objective of CKD management is to slow down disease progression. In the initial stages (1 and 2), strict control of comorbidities, including cardiovascular disease (CVD), is pivotal. In stages 3–5 of CKD, as complications arise, they must be systematically addressed [9]. The relationship between aging and the progression of CKD is complex and multifaceted, involving structural, hemodynamic, cellular, and metabolic changes. As the aging population continues to grow, a deeper understanding of these processes is crucial for developing targeted interventions and personalized treatment strategies to mitigate the impact of aging on CKD progression. Advances in this field hold the potential to enhance the quality of life for elderly individuals affected by this challenging and prevalent medical condition.

## 3. Hypertension in CKD and Dietary Salt Intake

High blood pressure that is not controlled can significantly increase the risk of CKD progressing to end-stage renal disease, cardiovascular disease (CVD), and mortality [22] and could represent a risk factor also for renal surgery functional outcomes [23]. Salt-sensitive hypertension is the hallmark of hypertension in CKD patients, where sodium reabsorption and volume excess are the fundamental mechanisms underlying it. CKD can be worsened by high salt intake [24,25,26]. This is because it causes volume expansion and glomerular hyperfiltration, leading to glomerular hypertension and ultimately focal glomerulosclerosis. As a result, patients lose nephrons at an accelerated rate. Additionally, salt affects the production of TGF beta, an inflammatory cytokine that promotes renal and myocardial fibrosis. In patients with CKD, the extracellular volume increases as the GFR decreases. In other words, there is an inverse relationship between extracellular volume and GFR. A post hoc analysis of the REIN 1 and 2 trials [27] studied the effect of salt on proteinuric CKD patients using different salt dosages: low salt (<125 mMol/day), medium salt (125–250 mMol/day), and high salt (>250 mMol/day). The study found that salt intake significantly impacted an important outcome, that is, the progression of nephropathy, which is a crucial outcome. Dietary salt intake can contribute to the progression of nephropathy, but it is a modifiable risk factor. This notion was based on a double-blind, placebo-controlled, randomized cross-over trial [28]. McMahon EJ et al. investigated the effects of dietary sodium intake on blood pressure, proteinuria, arterial rigidity, and extracellular volume in stages 3–4 CKD patients [28]. Their study found that reducing salt intake significantly reduced 24-hour ambulatory blood pressure, extracellular volume, albuminuria, and proteinuria [28]. These effects were even more pronounced than those observed in patients without CKD. These results emphasize the potential benefits of non-pharmacological interventions, such as sodium restriction, on reliable markers of kidney disease, such as proteinuria. Vogt et al. also reported similar observations in proteinuric patients without diabetes [29], where the effect of sodium restriction (<90 mMol/day) on proteinuria was comparable to that of Losartan 100 mg in the same patients.

The harmful effect of high salt intake on the kidney can be explained by various mechanisms. High salt intake increases arterial pressure and proteinuria by activating the RAAS, a mechanism that attenuates the proteinuria-lowering effects of RAAS blockers. Moreover, high salt intake increases glomerular capillary pressure and reactive oxygen species and promotes the inflammation of local tissues and endothelial dysfunction. All these mechanisms increase proteinuria and deteriorate kidney function. In this perspective, and considering that microalbuminuria is an early biomarker of renal damage, it is important to note that the use of drugs active on the RAS system is appropriate in the microalbuminuria phase in normotensive diabetic patients [30] to prevent further clinical sequelae leading to CKD.

It is also noteworthy to speculate that, although there are no specifically designed studies focused on this special topic, the Mediterranean diet has beneficial effects on blood pressure as well as on diabetes, obesity, and cardiovascular disease, thus potentially yielding positive effects on CKD progression [31]. The Mediterranean diet also exhibits advantages specific to CKD, including a decrease in dietary acid load, enhanced microbiota, and diminished inflammation. Opting for a higher intake of vegetables, fruits, and whole grains, coupled with a moderate intake of animal products, aids in lowering blood levels of phosphorus and potassium. Restricting the intake of ultra-processed products correlates with decreased levels of sodium, potassium, and phosphorus. The Mediterranean diet pattern is recommended for CKD patients, with adaptations tailored to the specific stage of the patients concerned [32].

The exact mechanisms by which hypertension enhances the progression of chronic kidney disease (CKD) are not yet fully understood. However, it is widely accepted that managing hypertension is crucial to prevent or slow down the progression of CKD. Apart from limiting salt intake and following a balanced Mediterranean diet, hypertension can be treated with drugs, such as angiotensin-converting enzyme (ACE) inhibitors and angiotensin receptor blockers (ARBs) [33]. These agents are well-known for effectively lowering blood pressure and reducing proteinuria.

There is scanty evidence that the use of RAS inhibitors benefits patients with advanced CKD, and current guidelines do not provide specific advice on whether to continue or stop ACE inhibitors or angiotensin-receptor blockers for advanced CKD. In a multicenter trial in which patients with advanced and progressive CKD were randomly assigned either to discontinue or to continue therapy with RAS inhibitors, the discontinuation of RAS inhibitors was not associated with a significant between-group difference in the long-term rate of eGFR decrease [34]. Thus, although the use of RAS inhibitors has been found to slow the decline in eGFR in patients with mild or moderate CKD, the findings of this trial are consistent with the hypothesis that these drugs may not be as helpful in patients with advanced and progressive CKD. SGLT2 inhibitors are a class of drugs that lower blood sugar levels by preventing the kidneys from reabsorbing glucose. SGLT2 inhibitors reduce the progression of CKD and diminish the probability of heart failure and mortality in patients with both CKD and type 2 diabetes. Additionally, these inhibitors also exhibit protective properties in non-diabetic CKD patients [35].

Therefore, since salt intake is a strong and independent predictor of cardiovascular and renal events in CKD patients, reducing and maintaining a low salt intake is essential to maximize the beneficial effect of ACE inhibition on CKD progression. While further studies with longer intervention times and larger sample sizes are needed to confirm these benefits, sodium restriction should be emphasized in managing patients with CKD to reduce the risk of progression. An approach for the self-management of salt intake could theoretically help in improving hypertension control, clinical outcomes, and autonomy in CKD patients. Indeed, the magnitude of the issue of high salt intake in CKD patients is similar to that of blood glucose control in diabetics. To this aim, a multicenter trial where hypertensive CKD patients self-monitored their salt intake was performed but with somewhat inconclusive results [36]. In addition to the mechanisms involved, from a clinical perspective, the main problem with hypertension in CKD is that it is often difficult to treat. Despite receiving treatment with three different anti-hypertensive drugs, including a diuretic, about one-fourth of CKD patients still have uncontrolled hypertension. It is important to note that this group of patients with CKD and resistant hypertension is characterized by a higher expansion of extracellular volume and a higher risk of cardiovascular and renal events [37]. Nocturnal hypertension is the most significant component of hypertension in CKD. It is defined as night-time blood pressure (BP) above the goal of 120/70 mmHg or as non-dipping status. These fundamental alterations in the circadian BP profile have a combined prevalence of up to 60% and represent a stronger predictor of poor cardiorenal outcomes [38,39]. Patients with resistant hypertension have 9% higher night-time BP levels compared with patients with other hypertension categories, while daytime BP differs by only 4% [40]. Recent analyses revealed a correlation between 24-hour urinary sodium excretion and night-time BP in patients with CKD [41]. One of the main risk factors for nocturnal hypertension in CKD patients is sleep apnea, which is mainly caused by volume overload [42,43]. However, this critical and often overlooked risk factor is not within the scope of the present review.

## 4. Obesity and CKD

Obesity is a worldwide epidemic, although the prevalence of overweight and obese people is geographically very different among countries and continents [44]. In fact, while in the USA, it is close to 70%, in the European community, the figures are somehow lower than in the American continent [45], but they are still a matter of great concern for the health organizations in European countries. The prevalence of severe obesity in Europe ranges from about 7% in France and Italy to 12% in Central Europe, and it climbs up to 20% in Eastern Europe and Russia [46]. The main concern about this epidemic is that several epidemiologic studies documented that obesity is associated with CKD and end-stage kidney disease. In other words, it is a risk factor for the onset and progression of CKD [47]. Moreover, to reinforce the opinion about the link between CKD and obesity, obesity and CKD go along in a strong parallelism, at least in the Western world. Considering the vast magnitude of the problem at hand, investigating the correlation between obesity and the CKD epidemic is an issue of paramount significance. Numerous epidemiological studies, including surveys and cohort studies, have provided evidence of a significant correlation between obesity and risk for CKD. Among these studies, the comprehensive investigation conducted by Hsu and colleagues on a large and diverse population in the United States was the largest to date [48]. Their study showed a clear and notable trend, indicating that the risk of CKD was directly proportional to the body mass index (BMI) of the participants. Specifically, the group with the highest BMI, classified as having very severe obesity, was found to have a seven times higher risk of CKD than the reference group with a normal BMI [48]. The relationship between obesity and CKD is intricate. While obesity is a recognized risk factor for CKD, the etiological pathway is not straightforward. Hypertension and diabetes, both common comorbidities of obesity, are established risk factors for CKD and are important contributors to the development of renal disease in obese individuals. However, the underlying mechanisms of this relationship are multifactorial and require further investigation. The Framingham Heart Study revealed that the waist-to-hip ratio (WHR), as a measure of abdominal obesity, is a more reliable predictor of CKD development than BMI [49]. Interestingly, the risk of CKD rises linearly across a range of WHR values between 0.7 and 1.2, independently of the Framingham risk factors, such as diabetes, hypertension, and other risk factors [49]. This independent effect brings into question the role of other risk factors in CKD development. Moreover, hemodynamic changes in the kidney occur before individuals develop frank obesity. Within a range of values below frank obesity, a significant decline in renal plasma flow can be observed with increasing body mass. However, this decrease is not accompanied by a simultaneous reduction in glomerular filtration rate (GFR), resulting in an increase in filtration fraction (the ratio of these two measurements) with higher BMI values [49]. Insulin resistance is a crucial factor in the hemodynamic adaptations of our body and a significant inverse correlation between the filtration fraction, an indicator of glomerular hyperfiltration/hypertension, and the glucose disposal rate, a measure of insulin sensitivity was clearly documented [50]. These results provide robust evidence that, at least in acute situations, insulin resistance can have detrimental hemodynamic effects on the glomeruli. Insulin resistance and hyperinsulinemia are well-known stimuli for sympathetic activity, which is associated with an increased risk of renal disease. Sympathetic activation can lead to renal disease through two mechanisms: mediated by systemic hypertension and direct. The increase in blood pressure caused by high sympathetic activity leads to nephroangiosclerosis, characterized by the simultaneous presence of ischemic and hyperperfused glomeruli. Additionally, sympathetic activity stimulates the synthesis of Angiotensin 2 and Aldosterone, resulting in volume expansion. This expansion further aggravates hyperfiltration in hyperperfused nephrons, particularly when salt intake is high, which is often the case in obese individuals. Ultimately, all these alterations lead to nephron obsolescence. The waist-to-hip ratio, a surrogate marker of visceral obesity, has been shown to independently predict the development of CKD [51]. This finding suggests that visceral fat may play a pivotal role in the pathogenesis of CKD. Visceral adipose tissue is known to secrete several biologically active substances, such as plasminogen activator inhibitor 1, leptin, adiponectin, angiotensinogen, and classical cytokines, that have the potential to cause renal damage [52]. Further investigations are warranted to evaluate the role of these and other risk factors in mediating the association between visceral obesity and renal damage in individuals with obesity. In the context of cardiovascular and renal damage, there is a range of mechanisms that can act either independently or via a common pathogenetic pathway such as endothelial dysfunction. Specifically, factors like insulin resistance, high sympathetic activity, and inflammation can have a deleterious impact on endothelial vasodilation, causing the synthesis of adhesion molecules that initiate the pro-atherogenic process. Obese individuals tend to have a reduced response to acetylcholine [53], but this response can be restored to normal levels with weight loss. One of the key indicators of obesity is endothelial dysfunction. Insulin resistance is the primary factor contributing to endothelial dysfunction [54]. Asymmetric dimethylarginine (ADMA), the most potent endogenous inhibitor of nitric oxide, is closely linked to both endothelial dysfunction and insulin resistance. Moreover, ADMA has been found to be involved in the progression to ESKD [55]. There is a growing body of evidence suggesting that weight loss can have a positive impact on kidney function. For instance, the Prevend study, which was a population-based study, found that a weight loss of 10 kg resulted in a decrease of 6 mg/24 h of microalbuminuria, which was independent of other factors [56]. It is worth noting, however, that this study was observational in nature and thus does not provide conclusive evidence about the efficacy of medical treatment policies. Despite the potential benefits of weight loss, the optimal strategy for preventing or halting the progression of renal disease in obese patients remains an open question. To date, there has been no controlled trial testing the effect of weight loss on renal disease progression, nor has there been any tested public health policy aimed at preventing CKD by controlling obesity. One potential approach to counteracting the renal hemodynamic dysfunction of obesity is the use of ACE inhibitors. However, to date, there has been no specific trial testing the effect of these drugs on renal disease progression in obese patients. To shed light on this issue, a secondary analysis of the REIN database within the framework of GISEN (Gruppo Italiano di Studi Epidemiologici in Nefrologia) was performed [57]. This analysis evaluated the efficacy of ramipril in patients based on BMI categories, with patients with less than 25 BMI being lean, those between 25 and 30 being overweight, and those with more than 30 being obese. The unexpected results were that ramipril had a beneficial effect in all BMI categories, particularly so in overweight and obese patients [57]. Furthermore, this effect was more pronounced after data adjustments for age and other risk factors. Overall, this secondary analysis supports the hypothesis that glomerular hypertension is implicated in the high risk of CKD in obese patients. Moreover, weight loss can also improve kidney function in people with CKD. A systematic review and meta-analysis of 13 randomized controlled trials found that weight loss interventions, including dietary modification, physical activity, and bariatric surgery, were associated with significant improvements in the estimated glomerular filtration rate (eGFR) and proteinuria in individuals with CKD [58]. However, it is important to note that weight loss should be gradual and sustainable, as rapid weight loss can potentially harm the kidneys.

## 5. Sympathetic Overactivity and CKD

Over the past decades, various papers have shed light on the close interplay between the sympathetic nervous system and CKD progression. Sympathetic overactivity is characterized by increased levels of norepinephrine and adrenergic receptor activation, which in turn act as triggers to exacerbate CKD. Furthermore, mounting evidence suggests that sympathetic overactivity is not limited to hemodynamic changes but also involves intricate inflammatory and fibrotic pathways [59]. The release of pro-inflammatory cytokines and the activation of fibroblasts contribute to the development and progression of renal fibrosis, a hallmark of CKD [60]. Sympathetic overactivity may act as a catalyst in these processes, exacerbating renal structural damage [61]. Understanding the impact of sympathetic overactivity on CKD progression has important clinical implications. Therapeutic interventions targeting sympathetic overactivity, such as beta-blockers and renal denervation, have shown promising results in clinical studies. These interventions aim to minimize the deleterious effects of sympathetic activation on renal hemodynamics and translate into benefits in terms of slowing the progression of CKD. The sympathetic nervous system plays a crucial role in modulating arterial pressure [61], renal blood flow, and sodium balance [62]. Elevated sympathetic activity contributes to =vasoconstriction of renal blood vessels, reduced renal blood flow, and activation of the renin–angiotensin–aldosterone system, which are all factors contributing to the deterioration of renal function over time. Vasoconstriction due to sympathetic overactivity increases glomerular pressure and acts as a pro-oxidative and pro-inflammatory factor [63]. In patients with renal disease, sympathetic overactivity increases cardiovascular risk and plays a primary role in the pathogenesis of hypertension [42,64,65,66,67]. Various papers show that hypertensive patients with mild renal impairment display a higher sympathetic activity as compared with hypertensive patients with normal renal function [68], and longitudinal studies reveal a close relationship between sympathetic overactivity and renal function deterioration over time [69,70]. Furthermore, accumulating evidence suggests that renal sympathetic nerves should be considered as a therapeutic target to reduce blood pressure and prevent the progression rate of renal impairment in CKD patients. Given the primary role of sympathetic overactivity in patients with hypertension and those with CKD, and the close association between sympathetic overactivity and adverse cardiovascular outcomes, therapeutic targeting of the sympathetic nervous system is expected to produce benefits in terms of clinical outcomes. The main therapeutic approaches include the blockade of peripheral β-receptors and the direct interference with renal afferent and efferent nerves via renal denervation [67]. The use of beta-blockers reduces the risk of cardiovascular events in patients with CKD and heart failure, but it is still uncertain whether such a beneficial effect is primarily due to the vasodilatory or antiarrhythmic properties of this drug class. On the other hand, recent studies on beta-blocker use in CKD patients show that about 25% of CKD patients are prescribed beta-blockers [71,72], and this limited use is in part due to the tolerability of these agents. In fact, it is well-known that beta-blockers may reduce cardiac output and the pressure of renal perfusion, thus worsening renal impairment [73]. The K/DOQI clinical practice guidelines suggest utilizing beta-blockers as the third-line antihypertensive option in patients with proteinuria [74]. Controversial findings regarding the renal-protective effects of atenolol in comparison to other antihypertensive agents have been reported in hypertensive patients with CKD [75,76]. In AASK Trials, no significant differences in a composite endpoint of renal outcomes and/or death were observed between patients treated with metoprolol and those treated with amlodipine [77]. Additionally, two studies highlighted the renal protective effects of carvedilol, a vasodilatory beta-blocker [78,79]. Thus, the notion that beta-blockers, in particular vasodilatory beta-blockers, should be considered as a third-line antihypertensive treatment for hypertension in CKD patients remains inconclusive. Finally, the HEMO study indicates a tendency toward the advantageous effects of beta-blockers for reducing sudden cardiac death in CKD with coronary heart disease but not in those without coronary heart disease [80]. Renal denervation directly interferes with efferent and afferent nerves linking the kidneys to the central structures in the brain. The resulting effect is the suppression of sympathetic outflow and eliciting protective effects in hypertension and heart failure. In patients with resistant hypertension, renal denervation reduces blood pressure as well as the activity of sympathetic nerve fibers [81]. Renal denervation has been consistently shown to be safe in various studies, and it holds the potential for particular advantages in patients with hypertension and CKD [81,82]. The benefits of renal denervation go beyond its effects on blood pressure. In a study including patients with resistant hypertension who underwent cardiac magnetic resonance before and after renal denervation, investigators not only observed a marked BP decrease but also a reduction in left ventricular mass and an increase in left ventricular systolic function [83]. Furthermore, although renal denervation has been used mainly to treat resistant hypertension, it is likely to also be beneficial in CKD. Indeed, renal denervation has been demonstrated to be associated with GFR improvement [84] and a reduction in albuminuria [85] in studies with a short follow-up period. Unfortunately, whether renal denervation has a potential effect on prognosis in CKD still remains to be formally investigated.

## 6. Conclusions

The interrelationship between CKD and aging, obesity, hypertension, salt intake, and sympathetic overactivity is complex and multifaceted. Addressing modifiable risk factors, such as maintaining a healthy weight, controlling blood pressure, reducing salt intake, and promoting a healthy lifestyle [86], can play a crucial role in preventing or slowing the progression of CKD. Thus, a comprehensive approach to managing these risk factors is essential in preventing and mitigating the impact of CKD on renal function. Regular monitoring, lifestyle modifications, and early intervention are key components of effective management strategies. Finally, given the fact that CKD progression is accompanied by increased cardiovascular risk, an approach contemplating multifactorial treatment aimed at reaching specific targets plays a primary role in reducing mortality and cardiovascular morbidity in the CKD population [87,88].
